# Development of a Course to Prepare Nurses to Train Expert Patients

**DOI:** 10.3390/healthcare13151939

**Published:** 2025-08-07

**Authors:** Manacés Dos Santos-Becerril, Francisca Sánchez-Ayllón, Isabel Morales-Moreno, Flavia Barreto-Tavares-Chiavone, Isabelle Campos-de Acevedo, Ana Luisa Petersen-Cogo, Marcos Antônio Ferreira-Junior, Viviane Euzebia Pereira Santos

**Affiliations:** 1 Department of Nursing, Federal University of Rio Grande do Norte, Natal 59078-970, Rio Grande do Norte, Brazilisabelle.campos@ufrn.br (I.C.-d.A.);; 2 Faculty of Nursing, Catholic University of Murcia, 30107 Murcia, Region of Murcia, Spain; fsayllon@ucam.edu; 3 Department of Nursing, Federal University of Rio Grande do Sul, Porto Alegre 90010-150, Rio Grande do Sul, Brazil; analuisa@enf.ufrgs.br; 4 Department of Nursing, Federal University of Mato Grosso do Sul, Campo Grande 79070-900, Mato Grosso do Sul, Brazil; marcos_junior@ufms.br

**Keywords:** health education, self-management of care, Primary Health Care, nurse, patient safety

## Abstract

Introduction: With the emergence of the expert patient and the expansion of health literacy, the importance of planning and building health technologies aimed at teaching and training health professionals, especially nurses, due to their activities with patients in Primary Health Care, with the aim of meeting the real and constant demands of the expert patient, is evident. Methods: Methodological study with a quantitative approach. The course was constructed based on a scope review, scientific reference, and observational visits during the months of September 2021 and August 2022. For validation, an organized electronic form was used with general information about the research and items of the course constructed for later evaluation by the judges with the three-point Likert scale and with the application of the Delphi Technique between the months of September and October 2022; for the agreement of the judges, the Content Validation Coefficient > 0.8 was considered. Results: Based on the content selected in the scope review, the reference contribution, and the observational visits, the course was constructed. Nine judges participated in the validation stage in Delphi I with a total Content Validation Coefficient above 0.90 and with some suggestions for modifications and improvements pointed out by them. In Delphi II, six judges evaluated the course, resulting in a total Content Validation Coefficient of 0.99. Conclusions: The course developed was considered valid to support the training of Primary Health Care nurses in the formation of the expert patient, with a view to promoting patient autonomy in self-care management, optimizing Primary Health Care, and reducing unnecessary hospital admissions.

## 1. Introduction

Expert patients are increasingly common, and their appearance and understanding are directly associated with the increased health literacy of this new group of health service users. These individuals are active in the self-management of their care, understand their own clinical conditions, and are more capable of solving simple daily issues associated with their health-disease process [1,2,3,4].

More and more, we find patients who acquire information from several sources, including television programs, the Internet, and mobile applications. They tend to share these findings with others who are in similar situations, building a network of support, coexistence, and solidarity to help one another [2,3,5].

Thus, it is important for health workers to be prepared to deal with these patients and work with them as well as possible, extracting and encouraging their positive characteristics, encouraging partnerships with the activities of the service that involve the community, and helping optimize the process of acquisition and analysis of new reports [1,6].

As a result, sessions to train health care centers workers and bring them up to date have been common. In these sessions, they develop abilities to identify, recruit, and train expert patients to improve the care provided, standardizing user-related activities, systematizing educational practices, and proposing plans to improve the health system [4,7,8,9].

It is worth noting that most professionals involved in these activities are nurses, seeing as this class comprises most of the workforce and continuously provides several direct care activities to their patients. They have increasingly become the main actors in this context, being autonomous and increasingly valued, offering courses related to health education from undergraduate level onward and working in many sectors and complexity levels [6,10,11].

Nursing also stands out due to its pedagogical side, which is proposed and encouraged in the training of these professionals and especially in the context of Primary Health Care (PHC), given that this level of care is considered to be the entryway to other services. The PHC receives a large number of people with chronic diseases and provides individual and collective educational activities to its users, being an extension of the social equipment in the community and the most accessible environment to deal with simple daily issues, related to population health [6,8,9,12,13].

However, although PHC is the most conducive context for identifying, recruiting, and training expert patients, nurses working there lack more information and/or training on how to carry out such activities. To solve this problem, international institutions in the northern hemisphere have developed educational strategies based on partnerships between educational centers and health services, with the aim of bringing together the different experiences and realities of academia and professional practice [1,3,4,8].

Thus, it is clear that one needs to plan, encourage, and build technologies for health professional training and education. This can reduce the shortcomings of their previous educational processes, bring their knowledge up-to-date with the fast and constant changes in many different contexts, strengthen health care practices, reduce additional costs within healthcare systems, encourage self-management of care among patients, and support the elaboration or revision of public policies, so they can be in line with the specificities of present time.

As a consequence, this study was made with the following guiding questions in mind: What should be the contents of a course to train PHC nurses to educate expert patients? Which elements should make up the structure of the course? Which theoretical, philosophical, and methodological aspects should be considered and/or be a part of the elaboration of the course? Our objective was to build and validate a course to train nurses in PHC so they can educate expert patients.

## 2. Method

This is a methodological, quantitative study, following Pasquali’s psychometric model [14]. This type of study is useful to produce and validate appropriate and reliable materials that can be used by other individuals [14].

### 2.1. Course Development

The course was created according to three domains: literature review; theoretical, philosophical, and methodological framework; and observational visits. Based on this information, the course was elaborated and organized with the following elements: characteristics, syllabus, objectives, content, methodology, evaluation, schedule, references, and appendices (Appendix A).

The literature review was, more specifically, a Scoping Review (ScR), and its research protocol was elaborated to guide the process of search and evaluation of studies. It was registered in the Open Science Framework (OSF) platform (DOI: 10.17605/OSF.IO/YPUVM) [15].

The following databases were consulted in January 2022: PubMed, CINAHL, SCOPUS, Cochrane CENTRAL, Web of Science, PsycINFO, Latin American and Caribbean Health Sciences Literature (LILACS), and Educational Resources Information Center (ERIC). In regard to the gray literature, we used the CAPES Thesis and Dissertations Portal, the National Library of Australia’s Trobe (Trove), the Academic Archive Online (DIVA), the DART-Europe E-Theses Portal, the Electronic Theses Online Service (EThOS), the Open Access Scientific Repository of Portugal (RCAAP), the National ETD Portal, Theses Canada, and Theses and dissertations from Latin America.

All materials about the study object that had been published online in full were included. We excluded editorials, books, opinion articles, and theoretical essays. There was no limitation with regard to time or language. The following variables were extracted from the works found: year of publication/defense; country of origin of the research; level of evidence; population/sample and profile of the study subjects; chronic disease; self-care actions; and main results.

The scoping review aimed to map existing educational strategies and essential skills related to the training of expert patients, in addition to the activities to be performed by nurses in identifying, recruiting, and developing this patient profile, so that these findings guided the better structuring, selection, and organization of the topics covered in the course.

Our philosophical, theoretical, and methodological references included the concept of Andragogy by Roque Luiz Ludojoski [16], David Paul Ausubel’s Theory of Meaningful Learning (TML) [17], and Albert Bandura’s Self-Efficacy Theory [18], respectively.

These authors were chosen because, although their reflections are different, their principles complement one another, as they allude and are compatible with the work of health professionals, since these individuals have needs, goals, experiences, and previous knowledge that are different from those of people who are still being formed as professionals.

In regard to the observational visits, they made it possible for us to observe the daily work in the institutions that develop activities involving expert patients. They were carried out in person and via remote meetings in Expert Patient Schools of Spanish autonomous regions and communities from September 2021 to May 2022, namely Cieza Health Center, Murcia Health Center, Granada Expert Patient Health Center, Valencian School of Health Studies, Seville Health Center, Madrid Health Center, and Mallorca Expert Patient Health Center.

Face-to-face and remote meetings were held at these Expert Patient Schools and in health units in the same regions that carried out initiatives and health care, educational, and managerial practices targeted at this new profile of service users. The observations also included meetings and discussions with professionals and/or patients of some of these centers; access to the materials produced there, such as videos and booklets; and others.

These moments were recorded through photographs previously authorized by those responsible for each center, based on conversations and informal reports during visits and continuous notes taken by researchers, as in a logbook. These measures helped to better understand the concept of expert patient, how the professionals involved in their formation are trained, what the educational methods are that are used, what the difficulties and potential for improvements are, and the positive results in the quality of life of users and for the health care services.

### 2.2. Validation Process and Selection of Judges

The content was validated using an electronic form produced in Google Forms^®^ (https://forms.gle/E84XmdUZpbHeutRp6; accessed on 10 July 2022), organized in the following topics: general presentation of the research, instructions for completing the questionnaire, virtual agreement to participate in the study, and sociodemographic and labor characterization. It also included the eight sessions that comprise the course, which were evaluated using a three-point Likert scale created according to an adapted version of the criteria proposed by Pasquali [14]. For each criterion in each session, a score of three indicated it was “adequate”; a score of two, partially adequate; and a score of one, adequate [19].

The content was evaluated by experts with some level of knowledge, contact, and/or professional experience in PHC, tracked in March 2022 via the Lattes Platform of the National Council for Scientific and Technological Development (CNPq), according to the criteria adapted from Fehring [20]: master’s degree in health (0 points), dissertation on PHC (1 point), research on the theme of self-care (3 points), article published in the field of PHC in a reference journal (3 points), doctorate in the field of health with a thesis on PHC (4 points), experience as a teacher (1 point), specialization in the field of PHC (2 points), totaling 14 points.

Immediately afterward, those who obtained a minimum score of up to 10 points were selected, a total of 60 judges, to whom an invitation was sent via email in order to explain the content of the survey and the importance of their participation.

In case of a positive response, another e-mail was sent with a consent form, in order to ensure that all ethical precepts were respected. After the consent form was sent back, signed by the evaluator, they were sent the evaluation form and asked to respond with the results in 20 days. For this process, a two-round Delphi Technique was used [21] from May to August 2022.

It is worth noting that, in Delphi II, in addition to the electronic form with the topics described above, judges were sent a file that included the suggestions for change given by them in the first round, as well as our justifications for accepting these suggestions or not. We excluded any specialists from the research who did not agree to participate, did not send back the signed consent form, or did not fill in the electronic questionnaire in Delphi I.

Data were tabulated and organized in Microsoft Excel 2010. We considered all items that reached an agreement of 80% among judges and a Content Validation Coefficient (CVC) > 0.8 valid [14,21].

This research is in accordance with the ethical precepts of Resolution No. 466, from 12 December 2012, by the National Council of Health. It was approved by the Research Ethics Committee at the Universidade Federal do Rio Grande do Norte (UFRN) under CAAE No. 46980621.2.0000.5537. It should be noted that all confidentiality criteria were met to ensure the anonymity of participants who agreed to take part in this study, so that judges were identified by the letter J (for judge) followed by an Arabic numeral according to the order in which their evaluations were received (J1, J2, J3, etc.).

## 3. Results

Using content from the ScR, the theoretical framework, and the observational visits, we reached the goal of creating a course to train PHC nurses, so they can educate expert patients. It was registered in the Ministry of Education Integrated Platform and is available at https://plataformaintegrada.mec.gov.br/recurso/360192 (accessed on 25 April 2023).

Content validation took place in two Delphi rounds, which took place from May to August 2022. Nine judges participated in Delphi I. Their sociodemographic and work characteristics are in Table 1.

Regarding their line of work, a single participant can work with more than one topic. Thus, they may be able to discuss the development of activities with the SP (09; 45.0%), health quality management (07; 35.0%), and health technologies (05; 20.0%).

As for the evaluation of the materials according to [14], there were agreement levels above 80% among judges in all items evaluated, with CVC values above 0.90 in Delphi I (Table 2).

After the first Delphi round, judges’ suggestions were considered to determine whether they should be included or not, considering the justifications shown in Table 3.

In general, the comments made by the judges aimed to clarify and enable a better refinement in the quality of the course’s structure, clarity, and content, mainly to assist in the flexibility for possible applications of this educational technology in other contexts and/or realities. In addition, all justifications and acceptances of the notes made were based on the theoretical, philosophical, and methodological references used throughout the course construction process.

After the changes were made according to the suggestions of the judges, a second Delphi round was conducted, and the same participants from the previous round were invited to participate. From the nine evaluators who participated in Delphi I, six participated in Delphi II. Their characteristics are in Table 4.

Regarding their line of work, a single participant can work with more than one topic. Thus, they may be able to discuss the development of activities with the SP (06; 40.0%), health quality management (05; 33.3%), and health technologies (04; 26.7%).

Regarding the evaluation of the materials according to [14], judges maintained agreement levels above 80% in all items evaluated, with a total CVC value of 0.99 in all items, except for the objectives (Table 5).

## 4. Discussion

The training of nursing professionals is increasingly more appreciated, given that people seek safe and good quality health care, making it necessary to increase the self-esteem and appreciation of these workers, helping change a hospital-centered practice and the curative-focused biomedical model [6,22].

This takes place, mainly, through the improvement and growth of PHC assistance, as it organizes and integrates health services according to the needs of the population. It also promotes the axes that structure the health system and are based on the care process, generating effective and efficient interventions [13,23].

PHC conditions have been extremely important in later years, especially in the post-pandemic world, since the number of people with chronic diseases increased exponentially. Furthermore, population aging and behavior and lifestyle changes contributed to the current epidemiological landscape [24,25].

In this regard, universities, technical education organizations, and health services from countries such as the United Kingdom, the United States, Canada, Australia, and Spain, have formed partnerships for the technical–scientific improvement in their health workers, so they can identify, train, and form expert patients, in order to provide continuous, holistic, preventive, and effective care to these individuals [5,26,27].

The search for different strategies to achieve the same goal is directly related to the multiple dimensions of patient care quality, including safety and effectiveness of care and patient-centeredness throughout the care process, so that healthcare professionals, especially nurses, show patients that they must be aware of their responsibility to themselves and be agents of change based on the results of their own actions [28,29,30].

These institutions often provide courses periodically, or according to existing or apparent needs, to improve the techniques used in the teaching-learning process; to produce and share scientifically-backed materials to optimize their activities; to create new national and international collaborative efforts to replicate successful activities; and to conduct investigations to evaluate the level of effectiveness of the educational practices being conducted [5,26,27].

Nonetheless, the motivation of nursing workers in regard to the contents and activities from the course is an essential element for individual or collective production. Active participation, together with the other subjects, is essential to raise prospective discussions and help significant learning [13,16].

To reach this result, the course must, in order to train health workers, be based on theoretical and methodological frameworks that are consistent with these individuals [16,17,18], appropriately using teaching strategies and tools with a direct connection to the contents addressed [5,27]. 

An example of this includes discussions in small or large groups about experiences with potential expert patients; mental maps to organize the understanding of the concept of expert patient; simulation scenarios that encourage the development of self-care actions and the resolution of daily problems, covered by patients and optimized by nurses; interactive quizzes and/or the collective elaboration of an interactive mural showing the benefits and the aspects that help or hinder self-care activities; and other possibilities [2,3,5,26,27].

In regard to the validation of the characteristics of the final sample in both Delphi rounds, the fact that most evaluators were female is a constant element in health throughout the years, especially in nursing. Furthermore, most participants are from this field, since it is the largest workforce in health services and is in accordance with the target audience of the course being elaborated [6,10].

In regard to their age group and time working in the field, although the Delphi II had 10 years less, participants were found to be experienced and have an affinity with the topics of this research, PHC, and safe care. This led to significant contributions that helped improve the quality of the structure and contents addressed in the course developed [11,12,13,22,30].

The same explanation can be associated with the time since the graduation of the judges, which varied from 10 to 20 years, and the fact that most worked with the triad teaching, research, and direct assistance. This indicates that the suggestions have a wide scientific basis, being closer to the issues found in the context of their work, whether this work is associated with scientific projects/articles or to the practices they carry out in health care units [13].

Nonetheless, the fact that most participants are associated with the topic of patient safety shows how important the topic is and how it must be considered in a cross-sectional way, from teaching to the activities developed by all those involved in the process of care—that is, health workers, patients/users and their families or support persons, based on the idea of providing a safer, higher-quality assistance [30,31].

Regarding the agreement level of the judges about the items evaluated, it was found to be uniform, as all items reached a total CVC above 0.90 since the first Delphi round. This indicates that the content was able to reach its goals. However, a Delphi II was necessary to consider the suggestions of the judges, which were relevant and increased the total CVC to 0.99 for all elements evaluated, with the exception of the objectives [32].

The suggestions proposed included clarifying the percentage of the course that was dedicated to in-person and remote activities and clarifying the essence of the approach and the goal of the course. Seeing as this is a type of training that requires developing and evaluating practical actions from the participants, in-person activities must be the most prevalent in order to provide a dynamic environment that can encourage the performance of these activities [23,33].

Additionally, given that the target audience is a portion of the public who work in the PHC for 40 h a week, it is important to make clear, since the beginning, that this course is a way to improve their work practices, causing no harm. Professionals and managers of the work units are contacted beforehand, so they feel more motivated and open to active participation in the course meetings [13,22].

In this sense, some strategies can be implemented jointly between health services, management, and educational institutions, such as establishing a fixed annual calendar of training and capacity building focused on the theme of the expert patient for these professionals; promoting the presentation of results obtained over time after the implementation of the activities suggested during the course and how this has impacted the quality of service; or even drafting public notices that encourage innovation in techniques to promote financial resources directed at this modality in patient care [11,23,28].

It is worth noting that helping promote a more receptive and interested behavior from course participants is valuable to reach positive results. This is in accordance with the Self-Efficacy Theory [18] recommended by a Delphi I judge and in andragogy, which discusses the education of younger adults, in this case, PHC nurses. The TML is also associated with this aspect of the research, as it generates a more robust cognitive structure by joining new and old knowledge [16,17].

This point meets the need to educate patients to be at the center of their care in an effective and safe manner, which is included as one of the teaching topics in the course and gives nurses a broader and more relevant understanding of the process of training expert patients in the context of PHC, with the aim of making them increasingly involved in self-management of care and safely assisting others in similar conditions [28,29,30,33].

Another idea from the judges was presenting and/or succinctly mentioning the concept of expert patients, which is the base of the course elaborated here, since the activities of the course aim to enable nurses to identify, recruit, and train this new patient profile. These patients would be able to self-manage their care, helping others in similar situations, have a more active attitude in regard to their own clinical conditions, and, although the topic is addressed during the meetings of the course, the target audience must have a general awareness about the contents to be addressed [1,2].

Concerning this suggestion, we would like to highlight the fact that this course is destined for PHC nurses because we want to encourage the community to recognize and use the PHC as the entryway to other health services. Moreover, there is a higher number of individuals who are potential expert patients, especially considering their characteristics, including having some chronic disease and being attended to in Primary Health Units [5,8,26].

Therefore, the relationship between PHC professionals and patients must be horizontal and based on trust and mutual responsibility, in order to bring short, medium, and long-term benefits to all those involved in the care process, even if there are some difficulties [6].

In general, all comments made by the judges were considered by the authors and promoted significant reflections, with the aim of improving the material developed and facilitating its applicability in other areas. In addition, even those observations that were redundant or refuted in Delphi II were given attention and, consequently, a plausible justification for not being accepted.

Limitations found in the development of this study include the impossibility of monitoring training sessions to form expert patients in the health centers and schools visited, since, at the time the visits were carried out, no session was being held; this prevented us from observing these sessions, which could help apprehend new ideas and experiences. Furthermore, the different understandings and experiences of the specialists during the evaluation process could generate mistaken interpretations.

Nevertheless, the results found here contribute to increasing the importance of seeing the patient as the center of their own self-care; to fostering reflections in nurses about the fast and constant epidemiological transitions and the need to update and adapt work strategies; to encouraging the building and validation of teaching tools for continued education in health services; and to increasing knowledge and, therefore, strengthen the activities carried out by health care professionals.

It is also intended to consider the construction and validation of this material as an initial subsidy for the elaboration of a practical action plan within the national health education program of the Ministry of Health, with the aim of overcoming cultural and linguistic barriers in a continental territory with wide epidemiological differences.

## 5. Conclusions

The course developed here to train nurses to educate expert patients was validated via judge evaluations. Although there were pertinent suggestions in the first Delphi round to improve the quality of the development of this technology, specialists gave a positive evaluation of the content. Therefore, the contents of the course were found to be adequate, meaning that the course was considered to be a valid way to support the training of nurses in the PHC, so they can educate expert patients, thereby promoting patient autonomy in self-care management, optimizing PHC care, reducing unnecessary hospital admissions, and encouraging the allocation of financial resources to other sustainable and necessary demands.

## Figures and Tables

**Table 1:** Sociodemographic and work characteristics of Delphi I judges.

Variable	n	%
Gender		
Female	08	88.9
Male	01	11.1
Age group		
30–40 years	02	22.2
41–50 years	04	44.5
51–60 years	03	33.3
Education		
Nursing	07	77.8
Pharmacy	01	11.1
Medicine	01	11.1
Time since graduation		
10–20 years	05	55.6
21–30 years	02	22.2
31–40 years	02	22.2
Field of work		
Research	01	11.1
Teaching and research	03	33.3
Teaching, research, and direct care	05	55.6
Time working in the field		
01–10 years	04	44.5
11–20 years	02	22.2
21–30 years	01	11.1
31–40 years	02	22.2

**Table 2:** CVC values in Delphi I.

Pasquali’s Criteria, Adapted	Characteristics	Syllabus	Objectives	Contents	Methodology	Evaluation	Schedule	Appendix A	Appendix B	Appendix C	Appendix D
Behavioral	0.99	0.96	0.96	0.99	0.96	0.96	0.99	0.96	0.96	0.96	0.96
Objectivity	0.99	0.96	0.92	0.96	0.99	0.96	0.96	0.99	0.99	0.99	0.99
Simplicity	0.99	0.96	0.96	0.96	0.96	0.99	0.99	0.99	0.99	0.99	0.99
Clarity	0.96	0.92	0.92	0.96	0.96	0.96	0.99	0.96	0.96	0.96	0.96
Relevance	0.99	0.92	0.96	0.96	0.99	0.96	0.99	0.96	0.96	0.96	0.96
Precision	0.99	0.96	0.92	0.96	0.99	0.99	0.96	0.99	0.99	0.99	0.99
Variety	0.96	0.99	0.99	0.99	0.99	0.99	0.99	0.99	0.99	0.99	0.99
Modality	0.99	0.96	0.99	0.99	0.99	0.99	0.99	0.99	0.99	0.99	0.99
Typicality	0.99	0.99	0.99	0.99	0.99	0.99	0.99	0.99	0.99	0.99	0.99
Credibility	0.99	0.92	0.99	0.99	0.99	0.99	0.99	0.99	0.99	0.99	0.99
Amplitude	0.96	0.96	0.96	0.96	0.99	0.96	0.99	0.99	0.99	0.99	0.99
Balance	0.99	0.99	0.96	0.96	0.99	0.99	0.99	0.99	0.99	0.99	0.99
Total CVC *	**0.98**	**0.96**	**0.96**	**0.98**	**0.98**	**0.98**	**0.98**	**0.98**	**0.98**	**0.98**	**0.98**

* refers to Caption: CVC = Content Validation Coefficient.

**Table 3:** Judge suggestions, modifications, and justifications after Delphi I.

JUDGES’ SUGGESTIONS	STATUS	RESPONSES TO JUDGES
**I—CHARACTERISTICS**		
Staff in Charge: Ddo. The Portuguese abbreviation is written incorrectly. I suggest rewriting it. Collaborators: PhD students, MS students, and graduation students (members of the research group). I suggest: Collaborators: PhD, MS, and graduation students (…)	Suggestion implemented	- We accepted the suggestion to describe the collaborators as PhD, MS, and graduation students (members of the research group)
I would just add the percentage of hours used for in-person and distance meetings.	Suggestion implemented	- The percentage of hours of in-person (80%) and remote (20%) meetings was described
**II—SYLLABUS**		
The concept of expert patient is not widespread and understood by all professionals	Suggestion implemented	- The word “concept” was added to the syllabus
CLARITY—since this is a new concept, I believe that a short and operational definition of the term “expert patient” should be presented in the documents of the course. I think that it must be clear to course students (nurses) what kind of behavior they will attempt to promote in these patients. I still did not understand clearly what I should expect (or not expect) from an expert patient. I do not know whether the syllabus is the best place for this, but I decided I should mention this in the beginning of the questionnaire.	Suggestion not included	- Since the target audience (Primary Health Care nurses) and the goal of the course already have a connection to the topic being discussed, and considering that it does appear in the main project from which this research stems (a thesis), the concept of expert patient was not detailed in the course documents. Nevertheless, in order to use this course in other contexts and with other audiences, we understand that it would be essential to present the goals of the course, including the definition of terms that are not much disseminated, as part of the announcements used to advertise the course.
(a) RELEVANCE—I believe that strategies to promote expert patients are more closely associated with other dimensions of quality (such as the centrality of the patient and effectiveness) than with safety—which is not to say that I disagree that an increased participation of the patient increases their safety. That in mind, I think that the beginning of the course should discuss the quality of care, as well as content on the centrality of the patient. As a consequence, the contents about safety could be more succinct. (b) An important point that, as far as I could see, was not mentioned in the course documents, is how professional-patient health relations are vertical, leaving little space for the patient to be active. I understand that in PHC this relation is less vertical than in hospital care, but still, one must consider the resistance of many professionals to recognizing and valuing the voice, the knowledge, and the experience of patients.	Suggestion not included	(a) We understand that the dimensions of quality management of care, such as patient centrality and effectiveness, are relevant and directly related to the goals of this course. However, since this study is part of a larger product focused on patient safety in Primary Health Care, and considering that said study found that there are shortcomings in this regard that should be addressed by the research subjects (with direct contact and participation in the main project), we chose to focus on patient safety and associate it with the patient expert in Primary Health Care. (b) The observation about the vertical nature of care and professional-patient relationships is also noteworthy, considering the culture of care that was built over time and the different realities that exist. However, although this is not clearly described in the syllabus, meetings 3, 4, and 5 will address evidence in regard to the aspects that make it easier or more difficult to train an expert patient in Primary Health Care, as well as the factors that help or hinder adherence to self-care, not to mention these topics were addressed in the theoretical context considered for the thesis.
The topic of the syllabus: The role of nursing in the health-disease process of chronic patients, is not closely connected to the goals and content suggested. You could make it more clear how this topic is associated with the context of the course.	Suggestion implemented	- The topic was changed for: The role of nurses and strategies to form expert patients.
**III—OBJECTIVES**		
(a) I think the objective “To analyze the benefits of expert patients, the aspects that hinder and help the self-care of chronic patients, discussing potential activities aimed at strengthening and minimizing them.” Cannot be reached, considering the content presented. I suggest removing it or transforming it in an object of the research, not of the training. (b) I also suggest including Bandura as a reference to support the training in regard to self-efficacy when adhering to self-care.	Suggestion implemented	(a) The objective was changed to: List the potential benefits of an expert patient, the aspects that make it more difficult and those that help the self-care of chronic patients, discussing potential actions in order to strengthen and minimize them. (b) Bandura was included as a reference in item V-METHODOLOGY, in addition to the references from Ludojovski and Ausubel.
Include the objective: 1. Train Primary Health Care nurses to develop expert patients. Include some communication tools and strategies.	Suggestion not included	- We believe that the objective suggested is the main objective of the course, since all objectives described in the course are the goals that the participants are expected to achieve, this includes becoming able to form expert patients.
(a) Patient safety is mentioned too often in the objectives of the course, with 4 out of 7 objectives being associated with it. In line with my previous comment, I think that one of the objectives could be related to the quality of care, and another, to the centrality of the patient. (b) An objective could also be included that is associated with the idea of the nurse “preparing” the health team, including physicians, to deal with patients that are more active in their health care.	Suggestion not included	(a) As explained above, although we understand the relevance of this association, this study is focused on patient safety in the PHC, and the objectives and initial content are associated with these topics. (b) The suggestion to include a goal related to the idea that the nurse could help/train the other team members in the training of the expert patient is included in the specific objectives of the 5th session of item IV—CONTENTS.
**IV—CONTENTS**		
A previous comment suggested reviewing the content in regard to the syllabus and its objectives.	Suggestion not included	- The non-inclusion was explained in previous Items
**V—EVALUATION**		
(a) I suggest making the attendance assessment clearer. 100% attendance? 75%? (b) Be clear about how active participation will be evaluated. (c) Will there be an instrument using indicators for this evaluation?	Suggestion implemented	(a) The minimum attendance will be 75% (in-person and distance meetings) (b) We indicated in item VI—EVALUATION which tools/activities will be used to evaluate active participation (c) No specific instrument will be used for this evaluation, which will be subjective
**VI—SCHEDULE**		
I think that, here, coherence with the objectives, as I commented above, is also relevant.	Suggestion not included	- The non-inclusion was explained in previous Items
**VII—APPENDICES (clinical simulation scenarios)**		
I would recommend changing terms that stigmatize the patient, such as “hypertensive”, to “person with systemic arterial hypertension”.	Suggestion implemented	- The term hypertension was changed into person with systemic arterial hypertension in the document as a whole.
**GENERAL COMMENTS**		
General comment: Since this course is targeted at health workers, and considering the difficulties in inviting and retaining participants in the course, I felt there was not sufficient information regarding how this aspect will be developed.	Suggestion not included	- Since this is a course, it is not possible to describe this aspect of the document. However, it explains that recruitment will be conducted using the existing means of communication (e-mail, phone, social networks, and others) to reach Primary Health Care nurses who are already close to the investigation at hand. Additionally, dates and times of meetings will be suggested and/or decided with the subjects beforehand.

**Table 4:** Sociodemographic and work characteristics of Delphi II judges.

Variable	n	%
Gender		
Female	05	83.3
Male	01	16.7
Age group		
30–40 years	01	16.7
41–50 years	02	33.3
51–60 years	03	50.0
Education		
Nursing	05	83.3
Pharmacy	01	16.7
Time since graduation		
10–20 years	03	50.0
21–30 years	01	16.7
31–40 years	02	33.3
Field of work		
Teaching and research	01	16.7
Teaching, research, and direct care	05	83.3
Time working in the field		
10–20 years	03	50.0
21–30 years	01	16.7
31–40 years	02	33.3

**Table 5:** CVC values in Delphi II.

Pasquali’s Criteria, Adapted	Characteristics	Syllabus	Objectives	Contents	Methodology	Evaluation	Schedule	Appendix A	Appendix B	Appendix C	Appendix D
Behavioral	0.99	0.99	0.99	0.99	0.99	0.99	0.99	0.99	0.99	0.99	0.99
Objectivity	0.99	0.99	0.99	0.99	0.99	0.99	0.99	0.99	0.99	0.99	0.99
Simplicity	0.99	0.99	0.99	0.99	0.99	0.99	0.99	0.99	0.99	0.99	0.99
Clarity	0.99	0.99	0.98	0.99	0.99	0.99	0.99	0.99	0.99	0.99	0.99
Relevance	0.99	0.99	0.99	0.99	0.99	0.99	0.99	0.99	0.99	0.99	0.99
Precision	0.99	0.99	0.99	0.99	0.99	0.99	0.99	0.99	0.99	0.99	0.99
Variety	0.99	0.99	0.99	0.99	0.99	0.99	0.99	0.99	0.99	0.99	0.99
Modality	0.99	0.99	0.99	0.99	0.99	0.99	0.99	0.99	0.99	0.99	0.99
Typicality	0.99	0.99	0.99	0.99	0.99	0.99	0.99	0.99	0.99	0.99	0.99
Credibility	0.99	0.99	0.99	0.99	0.99	0.99	0.99	0.99	0.99	0.99	0.99
Amplitude	0.99	0.99	0.99	0.99	0.99	0.99	0.99	0.99	0.99	0.99	0.99
Balance	0.99	0.99	0.99	0.99	0.99	0.99	0.99	0.99	0.99	0.99	0.99
Total CVC *	**0.99**	**0.99**	**0.98**	**0.99**	**0.99**	**0.99**	**0.99**	**0.99**	**0.99**	**0.99**	**0.99**

* refers to the Caption: CVC = Content Validation Coefficient.

## Data Availability

All data associated with our results are available upon request to the corresponding author and/or through the links made available throughout the article.

## Appendix A. PRE- AND POST-TEST

**Topic—multiple choice questions**

❖ ENERAL KNOWLEDGE ABOUT PATIENT SAFETY AND EXPERT PATIENTS

1. **According to the World Health Organization (WHO), Patient Safety means:**
(a) Promoting the best possible health care while reducing unnecessary harm to the patient
(b) Promoting health care with no harm to the patient.
(c) Promoting minimum health care while reducing unnecessary damage (related to human, scientific, and/or structural resources) to the patient.
(d) Promoting health care while reducing unnecessary harm to the patient to an acceptable degree.
2. **According to the national Program of Patient Safety (PNSP), published by the Ministry of Health (MH) through Ordinance No. 529 on April 1st, 2013, basic protocols for patient safety include:**
(a) Patient identification; hand hygiene; safe communication; fall prevention; pressure injury prevention; safe administration, and safe medicine use and administration.
(b) Patient identification; hand hygiene; safe surgery; fall prevention; pressure injury prevention; safe administration, and safe medicine use and administration.
(c) Patient identification; hand hygiene; safe surgery; fall and pressure injury prevention; safe communication; safe administration, and safe medicine use and administration.
(d) Patient identification; hand hygiene; safe surgery; fall and pressure injury prevention; safe communication; safe administration, and safe medicine maintenance, use, and administration.
3. **There are some factors that significantly help achieve satisfactory results when training expert patients, helping form the profile of an individual that actively manages their own care. Sign the incorrect alternative among the factors below:**
(a) The development of physical activities, seeking the integral wellbeing of the individual.
(b) Open and horizontal communication with health workers, from the perspective of a relationship based on trust and commitment between parties.
(c) Training/updating the knowledge of health workers, so they can be prepared to clarify doubts and adapt to the needs of patients.
(d) Active search for information about one’s clinical condition in reliable, scientifically backed sources.
4. **During the process of expert patient training, some actions are essential to ensure that the environment in which these experiences take place is the safest and most beneficial possible. They are:**
I. **Use icebreakers**
II. **Ask questions to a specific patient.**
III. **Recommend a periodic action plan**
IV. **Explain the diseases that affect the patients in detail**
V. **Promote group integration**
**Sign the correct option:**
(a) I, III, IV, and V are correct
(b) I, III, and V are correct
(c) II, IV, and V are correct
(d) I, II, III, IV, and V are correct
5. **Read the description below and sign the option that represents the most appropriate behavior in that situation:**
**An expert patient goes to a nurse’s office, asking some questions about a medication they use for arterial pressure control, after having found information about potential side effects this drug can have on its users.**
(a) Ask the patient to say what is the source of the information; report potential changes he observed in others; give orientations about how to find more reliable information about medicine on the Internet; research the drug in question; refer the case to the team’s physician.
(b) Ask the patient to say what is the source of the information; report potential changes he has been noticing/feeling; give orientations about how to find the most reliable information about medicine in general; research the drug in question; talk to the physician and/or interdisciplinary team about the case.
(c) Ask the patient to say what is the source of the information; report potential changes he observed in others; give orientations about how to find the most reliable information about medicine in general research the drug in question; talk to the physician and/or interdisciplinary team about the case; encourage the patient not to share the information with his family, to avoid worrying them unnecessarily.
(d) Ask the patient to say what is the source of the information; report potential changes he has been noticing/feeling; give orientations about how to find the most reliable information about medicine on the Internet; research the drug in question; talk to the physician and/or interdisciplinary team about the case; encourage the patient not to share the information with his family, to avoid worrying them unnecessarily.
6. **Although it demands time and dedication from those involved, the process of expert patient training brings benefits in the short, medium, and long term. Mark the options below as true (T) or false (F), considering these potential benefits:**
( ) **Improves one’s quality of life.**
( ) **Reduces costs**
( ) **Encourages multidisciplinary work**
( ) **Stimulates the development of problem-solving skills**
( ) **Involves learning about the culture of the individual**
( ) **Increases the reliability to identify and control the symptoms**
**Sign the correct option:**
(a) V-V-F-V-F-V
(b) V-F-F-V-F-V
(c) V-V-F-V-V-V
(d) V-V-V-V-F-V
7. **The professionals who are part of expert patient schools/programs use some tools and strategies to help identify individuals with the profile of expert patients. Select the correct option:**
(a) Clinical appointments and/or home visits
(b) collective activities
(c) Mobile applications
(d) All answers are correct
8. **The self-care of expert patients is complex, with both strengths and weaknesses, given that each patient has their own reality and specificities. The following are considered weaknesses:**
(a) Lack of motivation to change behavior, collective activities, health unit structural resources, comorbidities, focus on the disease, severity of the disease, physical limitations.
(b) Access to information, collective activities, health unit structural resources, comorbidities, focus on the disease, severity of the disease, drug accessibility.
(c) Lack of motivation to change behavior, environmental resources, financial resources, beliefs, severity of the disease, physical limitations.
(d) Access to information, collective activities, environmental resources, financial resources, beliefs, severity of the disease, drug accessibility.

## Appendix B. CLINICAL SIMULATION SCENARIO

**Subject**

▪ Identification/mapping of potential expert patients

**Objectives**

▪ General
○ To identify/map expert patients
▪ Specific
○ To discover and use the strategies/tools to search for and select expert patients
○ To develop and encourage multiprofessional work

**Length**

▪ A mean of 15 min

**Description:** the mean time described here refers to how long it takes to act out the clinical case, since the other stages of the simulation take their own time to be conducted. Course participants will be asked to participate in the clinical simulation voluntarily. For this clinical case, two volunteers will be necessary, while the others will watch the scene unfold. Everyone participates in the debriefing.

**Location**

▪ Meeting room of the Basic Health Unit

**Description:** The clinical skills laboratory will be adapted depending on the clinical case that will be simulated. In this case, it will represent a Primary Care Unit meeting room. This will be done to try and generate the closest setting to reality as possible, helping participants get involved in a natural manner.

**Scenario**

**Description:** The scenario includes the following elements: - Clinical case: brief description of the main situation, involving the issue being addressed in the case and the individuals involved; - Conduct: the commands (initial actions) given to individuals that participate in the clinical scenario, whether they are the main characters (volunteers) and/or are collaborating with the course (actors), so the scenario proposed can begin. - **Case evolution: refers** to the sequence of actions that the volunteers have to take during the case explained, which are different depending on the conduct they choose; - **Expected activities:** the set of activities that must or should be taken by the participants depending on the case at hand. They can be performed in full, partially or not at all, and will be observed and scored by the individuals who are not participating in the scenario at the time. During the debriefing, immediately after the case is concluded, the volunteers in the clinical simulation will have an opportunity to self-evaluate as to how well they did these activities.

▪ Clinical case
○ You are nurses from the Primary Healthcare Unit Soledade II. Since you have more experience in identifying and training expert patients, the manager of the service asked you to help a nurse that is new to the unit and has no knowledge at all about this type of conduct or patient profile. Thus, you will have to provide the necessary support, as you find pertinent.
▪ Conduct
○ The nurse who requested help should be informed of the reunion and invited to come to it with the other health workers, in order to learn how to identify an expert patient.

Collaborator in the role of nurse (actress): shows a shy, passive, and unquestioning attitude.

▪ Case evolution
○ Evolution of the situation presented in the clinical scenario, according to the actions of the nurse (collaborator/actress) and the responses/guidance given by the other nurses (volunteers).
▪ Expected activities

**Description:** These activities will be observed and scored by the participant observers and the facilitator throughout the development of the clinical case and later discussed in the debriefing.

**Action**	**Yes**	**Partial**	**No**
Volunteers had an open and cordial attitude			
Volunteers consulted the patients’ records			
Volunteers identified the epidemiological profile of the area of activity			
Volunteers used/managed the APRIDE correctly			
Volunteers suggested the participation of Community Health Agents to identify potential patients			
Volunteers organized a planned strategy to get in touch with the patients (home visits, clinical consultation, collective activities developed in the unit)			
Volunteers requested other professionals to participate (social workers, psychologists, dentists, nutritionists), providing different perspectives.			

**Human resources**

- Three nurses (one collaborator/actress and two volunteer participants)

**Material resources**

- A table
- Two chairs
- A4 paper sheets
- Ballpoint pens
- Computer
- Cellphone

**Debriefing**

**Description** For a broad discussion about what was done during the case and what the other participants observed, the debriefing is a fundamental stage to explain the situations experienced/observed. In it, all participants of the course, whether they participated in the scenario or just observed, can express their feelings, evaluations, and what they learned, in addition to any other elements they believe are helpful for the collective growth and to help clarify doubts. The questions listed below will help guide this stage.

▪ What was the order of the events? (Volunteers and observers)
▪ How did you feel during the meeting? (Volunteer)
▪ What were the positives, and what needs improvement? (Volunteers and observers)
▪ What would you do differently in another opportunity? (Volunteer)
▪ What did you learn from the experience with this simulated scenario? (Volunteers and observers)

**Evaluation**
The facilitator and the participants who were not in the simulation scenario will evaluate the events, describing the strengths and weaknesses they observed. All participants, except the facilitator, also have to indicate what they learned at the end of the simulation and at the end of the debriefing.

## Appendix C. CLINICAL SIMULATION SCENARIO

**Subject**

▪ Conducting the first meeting of expert patients

**Objectives**

▪ General
○ To teach a course for expert patients
▪ Specific
○ To identify the different patient profiles and their specificities
○ To encourage active communication among participants, especially the sharing of experiences
○ To plan and use appropriate techniques to conduct the activities of the course

**Length**

▪ A mean of 30min

**Description:** the mean time described here refers to how long it takes to act out the clinical case, since the other stages of the simulation take their own time to be conducted. The participants of the course will be asked to volunteer to act in the clinical simulation, keeping in mind those that did not have the opportunity to collaborate before. For this clinical case, one volunteer will be necessary, while the others will be observers. Everyone participates in the debriefing.

**Location**

▪ Meeting room for the Primary Healthcare Unit groups.

**Description:** the clinical skills laboratory will be adapted depending on the clinical case that will be simulated. In this case, it will represent a Primary Healthcare Unit meeting room. This will be done to try and generate the closest setting to reality as possible, helping the participants to get involved in a natural manner.

**Scenario**

Description: The scenario includes the following elements: - Clinical case: **brief description of the main situation, involving the issue being addressed in the case and the individuals involved**. - Conduct: the commands (initial actions) given to individuals that participate in the clinical scenario, whether they are the main characters **(volunteers) and/or are collaborating with the course (actors), so the scenario proposed can begin**. - **Case evolution: refers** to the sequence of actions that the volunteers have to take during the case explained, which are different depending on the conduct they choose; - **Expected activities:** the set of activities that must or should be taken by the participants depending on the case at hand. They can be performed in full, partially or not at all, and will be observed and scored by the individuals who are not participating in the scenario at the time. During the debriefing, immediately after the case is concluded, the volunteers in the clinical simulation will have an opportunity to self-evaluate how well they did these activities.

▪ Clinical case
○ You are the nurse responsible for conducting the first session of a course to train expert patients in a Primary Healthcare Unit. You will receive help from two community health agents, from two of the three fields of action of the health service at hand. The first session of the course will count on the participation of five patients who do not know each other, all of whom present with systemic arterial hypertension and/or diabetes.
▪ Conduct
○ The users (not entirely attentive) should be invited to form a conversation round in a large room with the community agents, where they will be informed about the motivation of the course and its goals.

Users (simulated patients—actors/students): have a confused posture, shy, not very communicative (at first). Community health agents (collaborators—actors): follow the instructions/questions of the nurse responsible for the course).

▪ Case evolution
○ The situation presented in the clinical setting will advance according to the actions of the users (simulated patients/actors) and the responses/orientations provided by the responsible nurse (volunteer).
▪ Expected activities

**Description: These activities will be observed and scored by the participant observers and the facilitator during the development of the clinical case.** Later, they will be discussed in the debriefing.

Action	Yes	Partial	No
The volunteer welcomes, receives, and requests the individual presentation of all components.			
The volunteer describes the importance of the course and its objectives			
Show empathy and personal interest in the users, based on their specificities (diseases, age, behaviors, and others)			
Use icebreakers			
Encourage users to share their perceptions and experiences			
The volunteer develops collective activities (pairs, trios) involving the course participants			
The volunteer clarifies user questions			
The volunteer explains, in a practical way, the concepts, signs, and symptoms of the diseases			
The volunteer recommends the main general and specific care activities			
The volunteer gives suggestions about different possible sources of information, encouraging active searches			
The volunteer asks the participants to suggest activities and/or topics to be discussed in the course			
The volunteer avoids value judgments and comments			

**Human resources**

- Two community health agents (played out by two collaborators/actors)
- Five patients (simulated patients—actors/students)
- One nurse (volunteer)

**Material resources**

- Chairs
- Paper
- Ballpoint pens
- Computer
- Projector
- *USB flash drive*
- Cellphone
- Glucose meter
- Blood glucose test strips
- Lancets
- Sphygmomanometer
- Stethoscope
- 70% alcohol.
- Cotton

**Debriefing**

**Description** For a broad discussion about what was done during the case and what the other participants observed, the debriefing is a fundamental stage to explain the situations experienced/observed. In it, all participants of the course, whether they participated in the scenario or just observed, can express their feelings, evaluations, and what they learned, in addition to any other elements they believe are helpful for the collective growth and to help clarify doubts. The questions listed below will help guide this stage.

▪ How did the participants feel during the first session of the course? (Simulated patients/actors)
▪ What was the order of the events? (Volunteers and observers)
▪ How did you feel while conducting the course? (Volunteer)
▪ What were the positives, and what needs improvement? (Volunteers and observers)
▪ What would you do differently in another opportunity? (Volunteer)
▪ What did you learn from the experience with this simulated scenario? (Volunteers and observers)

**Evaluation**
The facilitator and the participants who were not in the simulation scenario will evaluate the events, describing the strengths and weaknesses they observed. All participants, except the facilitator, also must indicate what they learned at the end of the simulation and at the end of the debriefing.

## Appendix D. CLINICAL SIMULATION SCENARIO

**Subject**

▪ The construction of an action plan for expert patients’ self-care

**Objectives**

▪ General
○ To elaborate an action plan for the self-care of the expert patient
▪ Specific
○ To list the elements that help or hinder adherence to the care plan
○ To trace strategies/actions that are in line with the reality and particularities of the patient

**Length**

▪ A mean of 30 min

**Description:** the mean time described here refers to how long it takes to act out the clinical case, since the other stages of the simulation take their own time to be conducted. The participants of the course will be asked to volunteer to act in the clinical simulation, keeping in mind those that did not have the opportunity to collaborate before. For this clinical case, one volunteer will be necessary, while the others will be observers. Everyone participates in the debriefing.

**Location**

▪ Nursing office at the Primary Healthcare Unit

**Description:** the clinical skills laboratory will be adapted depending on the clinical case that will be simulated. In this case, it will represent a nursing office in a Primary Healthcare Unit. This will be done to try and generate the closest setting to reality as possible, helping the participants to get involved in a natural manner.

**Scenario**

Description: The scenario includes the following elements: - Clinical case: **brief description of the main situation, involving the issue being addressed in the case and the individuals involved;** - Conduct: the commands (initial actions) given to individuals that participate in the clinical scenario, whether they are the main characters **(volunteers) and/or are collaborating with the course (actors), so the scenario proposed can begin**. - **Case evolution: refers** to the sequence of actions that the volunteers have to take during the case explained, which are different depending on the conduct they choose; - **Expected activities:** the set of activities that must or should be taken by the participants depending on the case at hand. They can be performed in full, partially, or not at all, and will be observed and scored by the individuals who are not participating in the scenario at the time. During the debriefing, immediately after the case is concluded, the volunteers in the clinical simulation will have an opportunity to self-evaluate how well they did these activities.

▪ Clinical case
○ After the first sessions of the course for expert patients, you, the nurse, start organizing individual consultations with the participants to create/review the action plan for the self-care activities of expert patients, according to the particularities of each of them. The current patient is Maria da Silva Oliveira (MSO), 40. She has systemic arterial hypertension, has smoked for 20 years, and has chronic obstructive pulmonary disease (COPD) and chronic kidney disease (CKD), which is why she has been receiving hemodialysis for the last two years. However, she has not been regularly present in her sessions. She is a housewife, lives with her retired mother, and is strongly religious, which is why she believes she will be cured by faith.
▪ Conduct
○ The user is invited to enter the nursing office, asked about her participation in the expert patient course, and what were its pros and cons.

User (simulated patient—actress): communicative, worried about her appearance, shows a positive attitude regarding her participation in the expert patient course, but always talks about faith as the solution to all her problems, including her clinical conditions.

▪ Case evolution
○ The situation presented in the clinical setting will advance according to the actions of the user (simulated patient—actress) and the responses/orientations provided by the nurse responsible for the consultation (volunteer).
▪ Expected activities

**Description: These activities will be observed and scored by the participant observers and the facilitator during the development of the clinical case.** Later, they will be discussed in the debriefing.

Action	Yes	Partial	No
The volunteer speaks cordially, stating how important the expert patient course and the action plan are			
The volunteer shows empathy and personal interest			
The volunteer promotes a safe environment, where a trusty conversation can be had			
The volunteer avoids value judgments and comments			
The volunteer actively listens to the patient			
The volunteer identifies elements that can interfere in the adherence to the care actions recommended			
The volunteer verifies how often the activities proposed are conducted			
The volunteer states that risks/consequences of not adhering to self-care actions			
The volunteer lists the self-care actions that must be followed			
The volunteer creates strategies to foster actions related to health care self-management			
The volunteers states the benefits that come from changing habits			

**Human resources**

- One patient (simulated patient—collaborator/actress)
- One nurse (volunteer)

**Material resources**

- Chairs
- Paper
- Ballpoint pens
- Computer
- Cellphone

**Debriefing**

**Description:** For a broad discussion about what was done during the case and what the other participants observed, the debriefing is a fundamental stage to explain the situations experienced/observed. In it, all participants of the course, whether they participated in the scenario or just observed, can express their feelings, evaluations, and what they learned, in addition to any other elements they believe are helpful for the collective growth and to help clarify doubts. The questions listed below will help guide this stage.

▪ How did the participant feel while conducting the nursing consultation? (Simulated patient/actor)
▪ What was the order of the events? (Volunteers and observers)
▪ How did you feel while conducting the consultation? (Volunteer)
▪ What were the positives, and what needs improvement? (Volunteers and observers)
▪ What would you do differently in another opportunity? (Volunteer)
▪ What did you learn from the experience with this simulated scenario? (Volunteers and observers)

**Evaluation**
The facilitator and the participants who were not in the simulation scenario will evaluate the events, describing the strengths and weaknesses they observed. All participants, except the facilitator, also have to indicate what they learned at the end of the simulation and at the end of the debriefing.
